# Converting a Common Low-Cost Document Scanner into a Multispectral Scanner

**DOI:** 10.3390/s19143199

**Published:** 2019-07-20

**Authors:** Zohaib Khan, Faisal Shafait, Ajmal Mian

**Affiliations:** 1 School of Information Technology and Mathematical Sciences, University of South Australia, Adelaide, SA 5095, Australia; 2 School of Electrical Engineering and Computer Science (SEECS), National University of Science and Technology (NUST), Islamabad 44000, Pakistan; 3 Deep Learning Laboratory, National Center of Artificial Intelligence, Islamabad 44000, Pakistan; 4 Department of Computer Science and Software Engineering, The University of Western Australia, Crawley, WA 6009, Australia

**Keywords:** multispectral imaging, document scanning, portable sensor

## Abstract

Forged documents and counterfeit currency can be better detected with multispectral imaging in multiple color channels instead of the usual red, green and blue. However, multispectral cameras/scanners are expensive. We propose the construction of a low cost scanner designed to capture multispectral images of documents. A standard sheet-feed scanner was modified by disconnecting its internal light source and connecting an external multispectral light source comprising of narrow band light emitting diodes (LED). A document was scanned by illuminating the scanner light guide successively with different LEDs and capturing a scan of the document. The system costs less than a hundred dollars and is portable. It can potentially be used for applications in verification of questioned documents, checks, receipts and bank notes.

## 1. Introduction

Forensic analysis of questioned documents involves a broad range of activities [1]. This includes establishing whether a document originated from a particular source, is backdated, forged or willfully manipulated. Disputes resolution over the authenticity of bank checks [2], purchase receipts, currency notes [3] or seals in agreements [4] can involve overwhelmingly complex legal procedures. In other cases, verification of the genuineness of the document source (written or printed) is also of significant importance to fraud detection [5]. The estimated age of a testament (will) can sometimes play a crucial role in the resolution of inheritance claims [6].

Traditionally, forensic scientists make empirical or experimental observations about a suspicious portion of the document in a forensic laboratory. The observations are then coupled with expert opinions to be presentable in a court-of-law. As this process largely relies on individual expertise and analysis, its consequences may be critical to the rights of a person, business or an organization. There is an interest in mechanisms for pre-examination of questioned documents before legally pursuing and bearing substantial costs in a court-of-law. Computerized forensic analysis has recently paved the way for automatic document forgery detection using *multispectral imaging* [7,8]. Multispectral or hyperspectral document scanners are generally comprised of bulky apparatus and require specialized laboratory environment for operation. This opens the need for the development of a portable multispectral document scanning system.

There are different ways of capturing multispectral images of a scene [9]. The most suitable method can depend on the target application. A *spatial scanner* simultaneously captures (*x*, *λ*) dimensions of a scene, whereas the *y* dimension is captured by the movement of the sensor or the scene. It is suitable for scenarios where either the scene or the sensing platform is moving such as in remote sensing. A flatbed multispectral document scanner can be regarded as a kind of spatial scanner, an example of which exists as a commercial device [10]. Flatbed scanners have a compact construction, however their scanning area is generally limited to an A4 size paper. Benchtop hyperspectral scanners have a similar operational procedure, and capture a relatively larger number of channels. The flexibility of a bench-top construction allows documents as well as other non-planar objects of interest to be scanned by the same device, at the expense of longer times per scan. Benchtop hyperspectral scanners have been shown to be useful for visual enhancement of old documents where a non-contact scanning mechanism may be preferred [11].

A *spectral scanner* simultaneously captures (*x*, *y*) dimensions of a scene, whereas the *λ* dimension is captured by spectral tuning [12]. It is specifically useful in a setup where both the scene and the sensing platform are stationary. The most common construction of a spectral scanner comprises a monochrome camera with a chromatic filter. A filter may be mechanically interchangeable using a wheel, which can be slow and require manual intervention. Such a device has been used for historical document image restoration [13]. A filter can also be tunable, thereby providing faster image scanning. The use of an accousto-optic tunable filter has been demonstrated for the purpose of document authentication [14] and liquid crystal tunable filter has shown to be effective in analysis of inks in documents [15]. However, camera captured documents suffer from image distortion due to perspective view, as well as non-uniformity of illumination. Moreover, the effective spatial resolution of a camera based multispectral document capture system may be much lower than a conventional document scanner.

In contrast, a *snapshot spatio-spectral sensor* simultaneously captures both spatial and spectral (*x*, *y*, *λ*) dimensions of a scene eliminating the need for scanning [16]. This method can effectively be used in conditions where the scene and the sensing platform are simultaneously moving. However, its complex sensor design incurs heavy costs limiting its use in applications such as in-vivo imaging of organisms [17].

Previously, we proposed a spectral scanning system for capturing multispectral images of a document [18]. Despite the simplicity of a static scene and the sensor, the system was prone to artifacts of camera captured imaging (illumination, perspective etc.) [19]. In this work, we propose a *spatio-spectral scanner* for capturing multispectral image of a document using a sheet-feed scanner, thus avoiding the problems associated with cameras. It captures one spatial dimension *x*, whereas the (*y*, *λ*) dimensions are sequentially acquired by feeding the document and tuning illumination spectrum, respectively. In the following section, we describe the proposed multispectral document scanning system, in terms of its electrical, spectral and optical design.

## 2. Materials and Methods

The main components of the proposed multispectral document scanner are an external multispectral light source and a standard document scanner.

### 2.1. Multispectral Light Source

A broadband source of light (e.g., incandescent or fluorescent) reflects the average response of a scene over a wide spectral range, and therefore achieves a low spectral fidelity. A multispectral source produces light in narrow spectral bands, attaining a high spectral fidelity. Light Emitting Diodes (LEDs) can provide such selectivity required in the spectral profile of a multispectral light source. Another favorable characteristic of LEDs is that they are highly energy-efficient compared to other sources of light.

#### 2.1.1. Electrical Design

**Figure 1:**
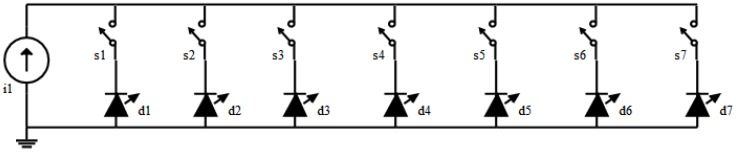
A schematic diagram of the multispectral light source showing the connection layout of the LEDs (d1–d7) and the constant current source (i1) via switches (s1–s7).

The electrical schematic of the multispectral light source is given in Figure 1. It consists of a constant current source (i1) connected to narrow-band LEDs (d1–d7) via switches (s1–s7). The constant current source limits the current from surpassing the absolute maximum current rating of the LED. It also makes an LED glow with the same luminous power and spectral profile, making the system reliable. However, an inadvertent connection of multiple switches simultaneously can result in the current being divided into several LEDs.

**Figure 2:**
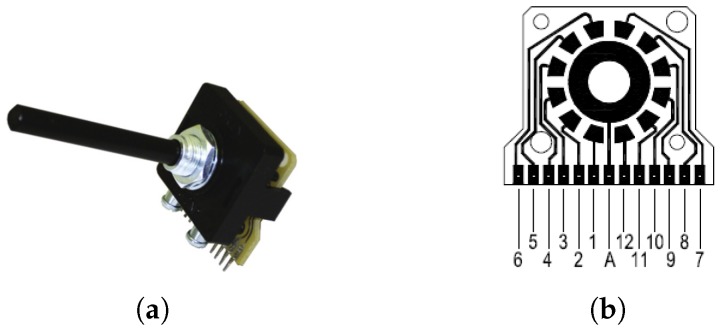
Schematic and assembled unit of a 30 degree indexing, 12 way unipolar switch: (**a**) *PT-6015* rotary switch from *Lorlin Electronics Ltd.*; Sussex, England and (**b**) schematic diagram of connection terminals.

To ensure only one LED is powered at a time, a unipolar multi-way rotary switch is included in the design. It provides non-shorting, break-before-make contacts, to avoid overloading of the source with multiple LEDs during switching. It can handle high currents of up to 500 mA @ 250 V ac/dc. The switch and its terminal positions as viewed from the knob end of the spindle are shown in Figure 2. Terminal A (middle) is connected to the positive end of the constant current source. Terminals 1–7 are connected to the positive terminals of d1–d7, respectively. If more spectral bands are desired to be captured, the corresponding LEDs can be conveniently connected to Terminals 8–12, which are currently not utilized.

**Figure 3:**
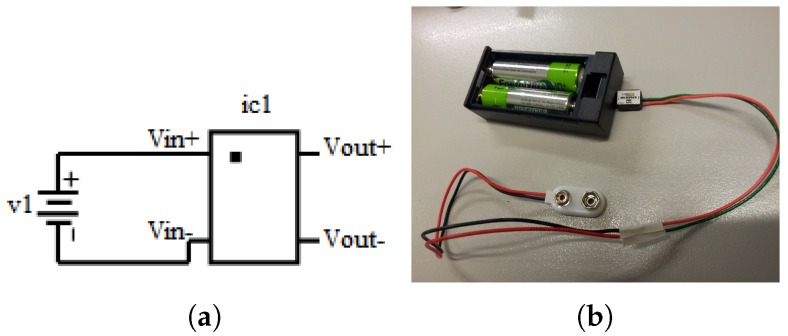

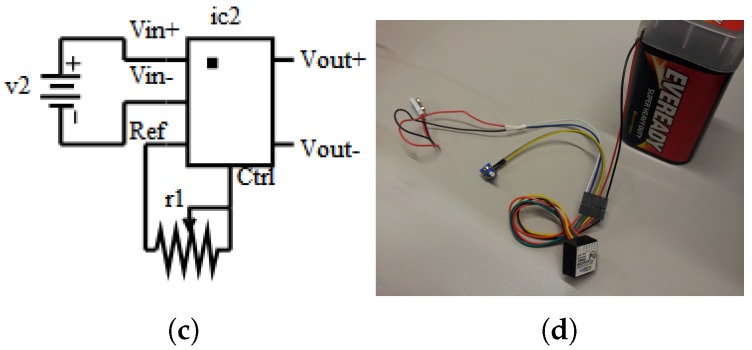
Schematic and assembly of constant current sources: (**a**) input terminals (V_in+_, V_in−_) of the MicroPuck driver (ic1) are connected to a low voltage source (v1 = 0.8–3 Vdc); (**b**) assembled low input voltage–constant current source; (**c**) input terminals (V_in+_, V_in−_) of the BuckPuck driver (ic2) are connected to a high voltage source (v1 = 7–32 Vdc) and the potentiometer (r1) allows dimming control (Ctrl) via internal reference (Ref); and (**d**) assembled high input voltage–constant current source.

Two constant current sources were designed depending on availability of a low or high input voltage source. The electrical schematic of the sources and their assembled form are shown in Figure 3.

The low input voltage–constant current source uses a MicroPuck LED Power Module which can provide a constant (350 mA) current to a single LED. The driver has two input pins (V_in+_, V_in−_) and two output pins (V_out+_, V_out−_). The miniature design allows use of one or two AA sized batteries to power the module. It provides the maximum current to the LEDs while mimicking the light drop-off of an incandescent bulb, which dims as the batteries drain. However, the current drops only at very low voltages, allowing maximum operational time.

The high input voltage–constant current source uses a BuckPuck LED Power Module which can provide a constant (350 mA) current to multiple LEDs. The module has four input pins (V_in+_, V_in−_, Ref, Ctrl) and two output pins (V_out+_, V_out−_). The module provides manual dimming control through a potentiometer which uses internal reference from the BuckPuck driver. It also has built-in protection for open-circuit and short-circuit.

#### 2.1.2. Spectral Profile

**Figure 4:**
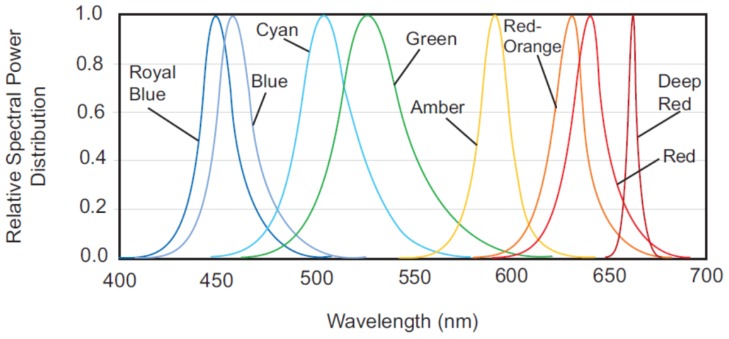
The relative spectral power distribution of the *Philips Luxeon Rebel* LEDs used in this study.

The choice of colored LEDs is important for description of the spectral profile of the multispectral light source. The spectral characteristics are characterized by two main parameters, i.e., the center wavelength and the spectral bandwidth. The relative spectral power distribution of the LEDs is given in Figure 4. These LEDs cover the majority of the range of visible electromagnetic spectrum (400–700 nm) at approximately regular intervals. The spectral parameters of the LEDs are provided in Table 1. Note that the LEDs are spread across the spectrum with sufficiently narrow-bands and high luminous power, which makes an effective multispectral light source.

Although the range of selected LEDs is in the visible spectrum, the proposed scanner design is generic and not restricted to the visible spectral range. Extension of the spectral range is a matter of adding LEDs (e.g., UV or infrared) in the proposed multispectral light source.

#### 2.1.3. Optical Configuration

**Figure 5:**
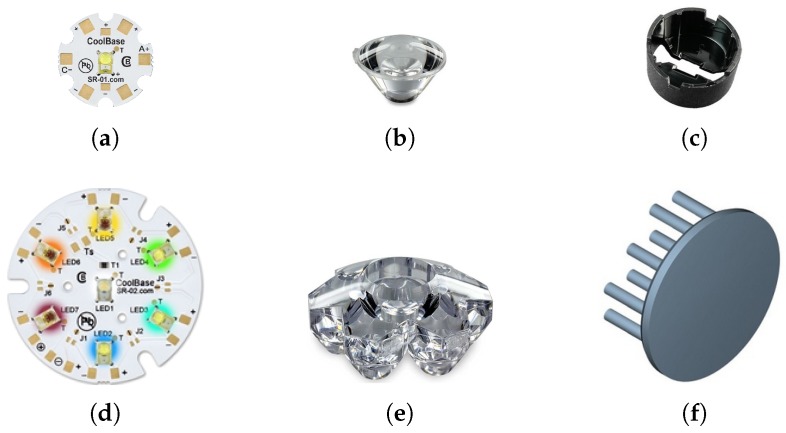
Components of the two optical configurations of multispectral light source: (**a**) single LED assembly; (**b**) fiber coupler concentrator lens; (**c**) circular lens holder; (**d**) seven-LED array assembly; (**e**) multi-cell cluster concentrator optic; and (**f**) natural convection heat sink.

**Figure 6:**
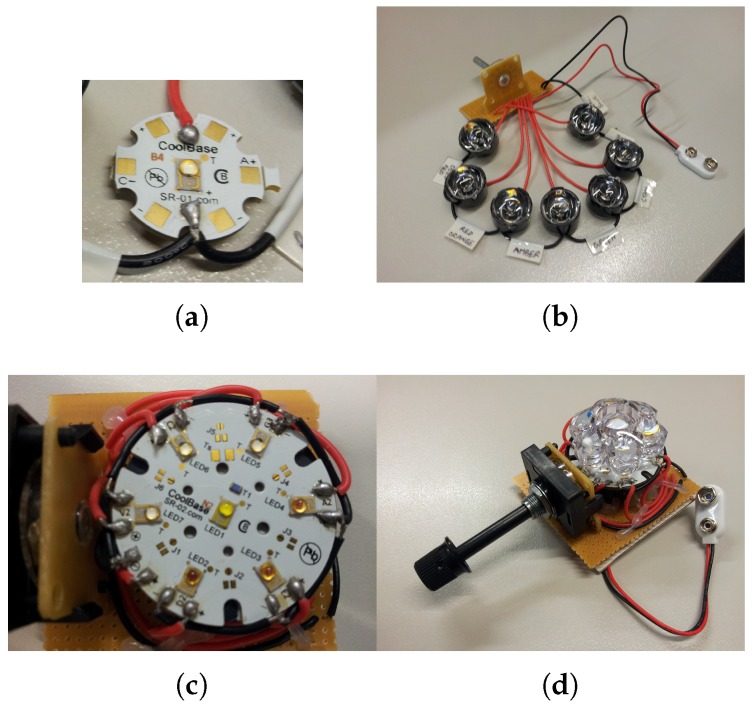
The assembly of linear and array optical configurations: (**a**) single LED base with connections to switch; (**b**) patched assemblies with fiber beam lens in linear configuration; (**c**) array LED base with connections to switch; and (**d**) patched assembly with cluster concentrator lens in array configuration.

The purpose of optical assembly is to transmit multispectral light into a flexible light guide, connected to the scanner light guide. Concentration optics are suitable for beam insertion into fiber optic bundles or light guides. Two different optical arrangements were proposed for multispectral light source, as shown in Figure 5. The two optical configurations after the assembly are shown in Figure 6.

In the linear arrangement, an LED is pre-soldered to a base with anode(+) and cathode(−) connections at the locations shown in Figure 5a. A fiber beam lens (*Carclo Optics, Aylesbury, England*), shown in Figure 5b, focuses light from LED into an eight-degree narrow beam at a focal distance of 11 mm. The diameter of the lens is 20 mm and conforms to the LED base. It requires a circular lens holder, as shown in Figure 5c, which is affixed to the base using a double-sided tape. The holder positions the lens at an appropriate distance from the LED to obtain the maximum luminous transmission. Multiple such units, each with a different colored LED, together make a multispectral light source.

In the array arrangement, multiple LEDs are pre-soldered to a single base with separate anode(+) and cathode(−) connections for each unit at the locations shown in Figure 5d. A cluster concentrator optic (*Polymer Optics Ltd.*), Berkshire, England shown in Figure 5e, focuses light from seven LEDs into a 12-mm narrow beam at a focal distance of 25 mm. It is made of an optical grade poly-carbonate material for thermal stability and system durability, which results in a high light collection efficiency (85%). The use of the array LEDs and the cluster optic makes the light source compact and rigid.

The use of high-power LED array can introduce significant overheating if it is not correctly catered for. A heat sink is an affordable device for maintaining near constant temperature of LEDs for long periods of operation. The *CN40-15B* heat sink from *ALPHA Co. Ltd.*, Shizuoka, Japan has a 40-mm round base with 15-mm legs, as shown in Figure 5f. It has the highest thermal efficiency in the *CN40* series of heat sinks.

### 2.2. Document Scanner

**Figure 7:**
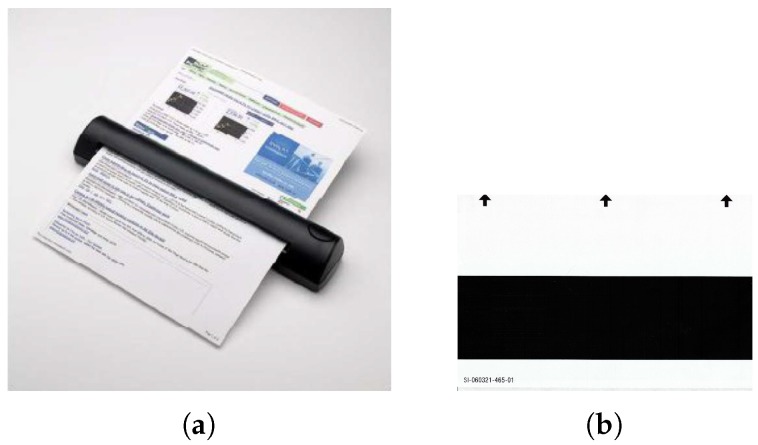
An automatic feed portable document scanner can be converted into a multi-spectral document scanner: (**a**) the *DSmobile600* sheet-feed portable document scanner from *Brother Mobile Solutions Inc.*, Westminister, CO, USA used in this work; and (**b**) a standard black and white reference sheet for calibration. Arrows indicate the direction of feeding into the scanner.

Connection of an external source of light can be intrusive to the movement of the scanner carriage unit in a flatbed scanner, which may cause discrepancies in the scanned image. In contrast, a sheet-feed scanner allows integration with an external source of light without being intrusive to the scanner operation. Since the scanning unit of a sheet-feed scanner is stationary, its operation is not affected by connection to an external source of light. Moreover, the size of a sheet-feed scanner is mainly determined by the shorter edge of the supported page size, which makes it compact and portable, as shown in Figure 7a. A sheet-feed mechanism is therefore preferred over a flatbed construction to form the basis of a multispectral document scanner.

#### 2.2.1. Scanner Modification

**Figure 8:**
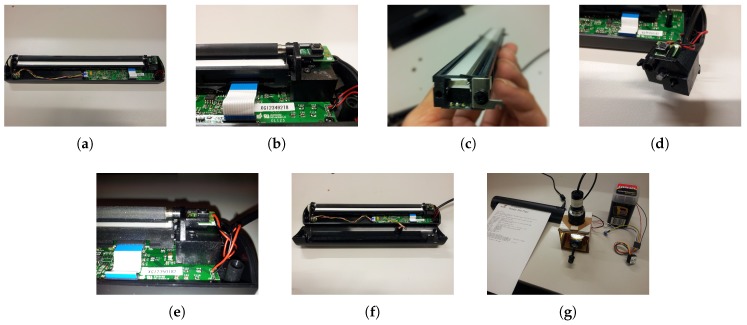
The steps of the scanner modification and its connection to the multispectral light source: (**a**) remove the top cover to gain access; (**b**) release the components from the hinge support; (**c**) disengage RGB LED from the scanner sensor; (**d**) make provision for a flexible light guide; (**e**) re-install the components; (**f**) replace the top cover; and (**g**) connect to the multispectral light source.

Modification of the sheet-feed scanner consists of the following steps (the procedure is illustrated in detail in Figure 8):

1. Gain access to the internal micro illumination source (RGB LED) of the scanner.
2. Remove/Disable the internal RGB LED.
3. Structurally modify the scanner housing for placement of a flexible light guide.
4. Connect the external light source to the internal light guide via the flexible light guide.

#### 2.2.2. Scanner Calibration

The multispectral scanner can be calibrated using a special black and white glossy sheet that came with the scanner, a sample of which is shown in Figure 7b. The bright and dark values of each spectral band can be computed using the scanned reference sheet and applying a formula for normalization:

(1) $$C\left(x,\lambda \right)=\frac{I(x,\lambda )-D(\lambda )}{B(x,\lambda )-D(x,\lambda )}$$

where *I* is the original image, *C* is the calibrated image, and *B* and *D* are the average bright and dark points at each wavelength, respectively.

### 2.3. Multispectral Document Scanning

**Figure 9:**
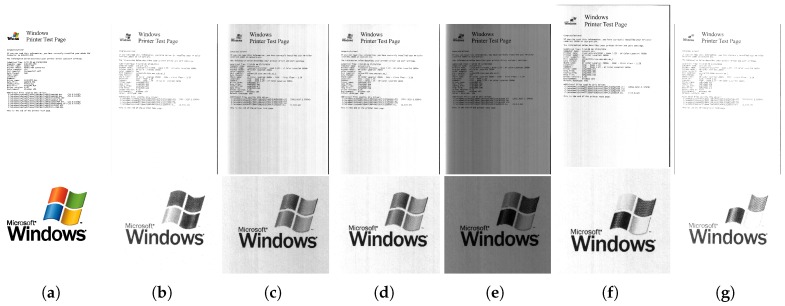
The printed test document (**top**) and a magnified view of the logo contained within (**bottom**) in: (**a**) true RGB; (**b**) royal blue; (**c**) cyan; (**d**) green; (**e**) amber; (**f**) red orange; and (**g**) deep red bands.

To test the multispectral document scanner, a test page printed from an HP Laserjet Color printer was scanned. The RGB true color image and various bands of a logo in the test page captured by the multispectral scanner are shown in Figure 9. A relative variation in the brightness can be observed between the bands due to the differences in spectral power and bandwidth of the LEDs. The explanation for a relatively darker scanned image using the Amber LED is its low spectral power coupled with a narrow bandwidth, which together cause a weaker response at the detector.

**Figure 10:**
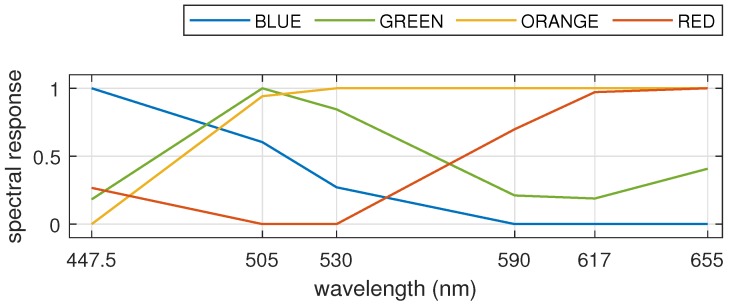
Normalized spectral response of the four colored elements in the Windows logo plotted against the center wavelengths of LEDs in the multispectral light source.

Observe that the logo has red, orange, green and blue elements and black text at the bottom. The normalized spectral response computed by averaging an 11 × 11 patch at the center of each colored element is shown in Figure 10. Notice that the different components of the logo have characteristic intensity response to multispectral light according to the spectral band. This demonstrates the ability of the scanner to capture fine details in the spectrum.

We further tested the developed prototype to identify counterfeit protection system (CPS) codes inserted in color print-outs by all consumer printers [20]. The recent availability of high-resolution printers has not only supported useful purposes, but also paved the way for illegal manipulation of documents. This has consequently persuaded color printer manufacturers to hide an invisible CPS code, which holds information for printer identification. This unique code is printed in every document, in the form of a repeated pattern of yellow dots that is not visible to the naked eye. The unique pattern can be used to identify the source of a document. The multispectral document scanner successfully captures this unique dot-pattern, which can be extracted by binarization of the raw image using image thresholding operation.

**Figure 11:**
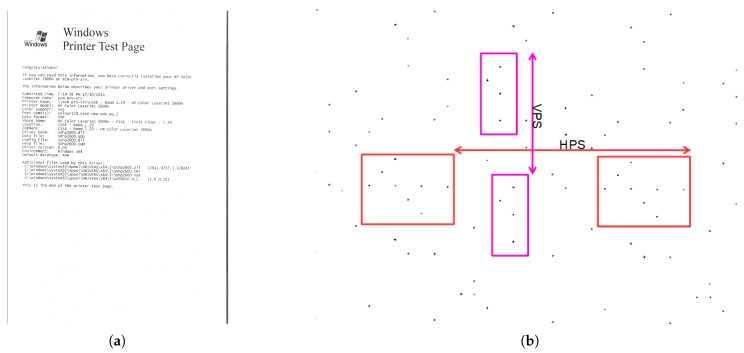
Extraction of counterfeit protection pattern by image processing: (**a**) Royal Blue band of a test printed document (zoom to 6400% to clearly view the pattern); and (**b**) processed image of enhanced codes separated by horizontal pattern separation (HPS) and vertical pattern separation (VPS).

The unique patterns of different printers can be identified in terms of their geometrical structures. The two important parameters that form these relationships are the Horizontal Pattern Separation (HPS) distance and the Vertical Pattern Separation (VPS) distance. A raw image of the Royal Blue band of the scanned test page and its patterns enhanced by image processing, which comprised thresholding and image binarization, are shown in Figure 11. The processed image is magnified to visually identify the recurring CPS code. The HPS and VPS measurements were then annotated in the processed image. The CPS code can now be extracted and analyzed using HPS and VPS measurements.

## 3. Conclusions

We present the design of a prototype multispectral document scanner, which is demonstrated to capture subtle features in a document using a multispectral light source. The multispectral light source was designed to cover the full range of visible electromagnetic spectrum and connected to a portable sheet-feed document scanner. This light source is comprised of commercial off-the-shelf LEDs of various wavelength, bandwidth and radiant power.

An optimal design may comprise a selection of custom-built LEDs for precise selectivity across the spectrum. These LEDs may also be designed to emit a fixed luminous flux and bring homogeneity in the brightness of bands. The addition of more LEDs will further enhance the capabilities of the device. For instance, the addition of an ultraviolet LED can enable the device to capture invisible security features hidden in some banknotes for verification. Similarly, the addition of an infrared LED can allow the device to capture forgeries in handwritten or printed text for question document examination.

The scanner was calibrated using a white–black reference sheet, which achieved normalization of spectral responses, albeit relative to each band. In circumstances where an absolute spectral response is necessary, the scanner would require calibration with the output of a spectrometer and validated on the same calibration reference. While it is sometimes important to measure absolute spectral response, many applications can simply benefit from a relative (normalized) spectral response measurement to achieve the intended results, as presented in the current system.

In regards to the scanner operation, currently the light source is switched to the desired color by means of a rotary switch, and one band of the document is captured in each pass. A more efficient operation can be built upon electronic switching of the multispectral light source in a time-multiplexed manner to capture all bands in a single feed. However, this modification would require changes to the scanner software to synchronize switching of the external multispectral light-source with the scanning of detector.

Given the presented system and proposed directions of improvement, the prototype design has the potential to be transformed into a fully functional portable device suitable for multipurpose document analysis.

## Figures and Tables

**Table 1:** Specifications of *Luxeon Rebel* series LEDs at 350 mA.

Color	Center Wavelength (nm)	Bandwidth (nm)	Flux or Power (lm,mW)	Part Number Model
Deep Red	655	20	360	LXM3-PD01
Red	627	20	48	LXM2-PD01-0040
Red-Orange	617	20	56	LXML-PH01-0050
Amber	590	20	48	LXML-PL01-0040
Green	530	30	95	LXML-PM01-0090
Cyan	505	30	76	LXML-PE01-0070
Blue	470	20	41	LXML-PB01-0040
Royal Blue	447.5	20	1030 ^‡^	LXML-PR02-A900
Neutral White	-	-	180	LXML-PWN1-0100

^‡^ tested at 700 mA.
